# Salivary glands of primary Sjögren's syndrome patients express factors vital for plasma cell survival

**DOI:** 10.1186/ar3220

**Published:** 2011-01-07

**Authors:** Ewa A Szyszko, Karl A Brokstad, Gunnvor Øijordsbakken, Malin V Jonsson, Roland Jonsson, Kathrine Skarstein

**Affiliations:** 1 Broegelmann Research Laboratory, The Gade Institute, University of Bergen, The Laboratory Building, Bergen, N-5021, Norway; 2 Section for Pathology, The Gade Institute, University of Bergen, Haukeland University Hospital, Bergen, N-5021, Norway; 3 Institute of Medicine - Section for Rheumatology, Haukeland University Hospital, Bergen, N-5021, Norway; 4 Department of Rheumatology, Haukeland University Hospital, Bergen, N-5021, Norway

## Abstract

**Introduction:**

The presence of circulating Ro/SSA and La/SSB autoantibodies has become an important marker in the classification criteria for primary Sjögren's syndrome (pSS). Plasma cells producing these autoantibodies are mainly high affinity plasma cells originating from germinal centre reactions. When exposed to the right microenvironment these autoimmune plasma cells become long-lived and resistant to immunosuppressive treatment. Since autoimmune plasma cells have been detected in the salivary glands of SS patients, we wanted to investigate if the glandular microenvironment is suitable for plasma cell survival and if glandular residing plasma cells are the long-lived plasma cell subset.

**Methods:**

Single, double and triple immunohistochemistry as well as immunofluorescence staining was performed on minor salivary gland tissue retrieved from pSS, chronically inflamed and normal subjects.

**Results:**

We detected significant numbers of CD138+, non-proliferating, Bcl-2 expressing plasma cells in the salivary glands of pSS patients with high focus score (FS). Furthermore, we demonstrated that CXCL12 and interleukin (IL)-6 survival factors were highly expressed in pSS salivary gland epithelium and by focal mononuclear infiltrating cells. Notably, adipocytes when present in the salivary gland tissue were an important source of CXCL12. We clearly demonstrate that plasma cells are localised in close proximity to CXCL12 and IL-6 expressing cells and thus that the environment of salivary glands with high FS provide factors vital for plasma cell survival.

**Conclusions:**

Plasma cells residing in the salivary glands of pSS patients with high FS showed phenotypic characteristics of the long-lived plasma cell subtype. Furthermore, the pSS salivary gland microenvironment provided niches rich in factors vital for plasma cell survival.

## Introduction

Sjögren's syndrome (SS) is a heterogeneous autoimmune disease characterized by focal mononuclear cell infiltration in the exocrine glands and high serum titres of Ro/SSA, La/SSB and rheumatoid factor (RF) autoantibodies. Plasma cells producing these autoantibodies are primarily class-switched, somatically mutated IgG plasma cells that origin from germinal centers (GCs) reactions [1]. However, detection of autoreactive plasma cells in the inflamed salivary glands [2,3] and presence of IgA autoantibodies in sera and saliva of SS patients [4-6] raise questions about the origin and contribution of salivary gland plasma cells to the pathogenesis of SS.

In addition to plasma cells, the focal infiltrates in salivary glands of SS patients consist of T-cells, B-cells, macrophages, follicular dendritic cells, dendritic cells and plasmacytoid dendritic cells [7-10]. In approximately one-fourth of patients with primary SS (pSS), the accumulating cells form structures that resemble GCs as seen in secondary lymphoid organs [11-15]. Together with the fact that both Ro and La antigens have been detected in the salivary glands of SS patients [16,17] there exists a possibility that autoimmune plasma cells are produced at the site of inflammation. Another possibility is that the glandular microenvironment comprises factors necessary for prolonged plasma cell survival and that autoantibodies are being produced by plasma cells independently of activation and differentiation of new B-cells. This phenomenon has been shown for the bone marrow residing plasma cells that in the presence of particular survival signals produce circulating immunoglobulins in an antigen independent fashion [18-24]. The bone marrow subset of plasma cells is often referred to as a long-lived plasma cell subset.

The importance of long-lived plasma cells in autoimmunity has been brought to light after observations made during clinical studies utilizing the B-cell depleting monoclonal antibody rituximab in systemic lupus erythematosus. As an outcome, anti-CD20-treated autoimmune patients showed drastic reductions in B-cell numbers, but unchanged serum levels of autoantibodies [25,26]. Thus, it has been proposed that at least some portion of these autoantibodies is being produced not by newly generated cells but by pre-existing long-lived plasma cells, unaffected by treatment.

In order to survive, long-lived plasma cells need contact with particular factors present in their environment. In the bone marrow these survival signals are provided by the so-called survival niches generated by bone marrow stroma [22,23,27-29]. Interestingly, many of the survival molecules in particular cytokines and chemokines, are also important modulators of the immune responses that occur within the inflamed tissues. In the salivary glands of SS patients, the mononuclear cell infiltrates are one of the hallmarks of the disease. However, it is the glandular microenvironment and stromal cells of the glands that are responsible for accumulation, location and retention of the inflammatory cells [30].

In the present study we aimed to investigate: I) If the microenvironment of the salivary glands of pSS patients provides factors necessary for plasma cell survival and if plasma cells are indeed in close contact with these factors; II) If plasma cells present in minor salivary glands of patients with pSS are potential candidates for the long-lived plasma cell subset; III) If the glandular microenvironment of pSS is disease specific and thus differs from the glandular microenvironment in non-SS tissue; IV) If GC-like structures have an impact on the type of microenvironment seen in minor salivary glands; and finally V) If the survival molecules contribute to the migration of plasma cells into inflamed salivary glands.

## Materials and methods

### Patients and controls

Lower labial minor salivary gland biopsies from 21 consecutive patients fulfilling the American-European consensus group criteria (AECC) of pSS [31] and 10 individuals not fulfilling the pSS criteria were used in this study. All biopsies had been performed between 2004 and 2007 at the Department of Otolaryngology/Head and Neck Surgery at Haukeland University Hospital in Bergen. All tissue sections had been previously evaluated by an oral pathologist and the focus score (FS) (the number of focal mononuclear cell infiltrates with >50 mononuclear cells per 4 mm^2^) was determined. All patients had FS ≥1 and were divided in two groups; patients with given FS equal to 1 (FS = 1) and patients with FS equal to or more than 2 (FS ≥2). Hematoxylin and eosin (H&E) stained formalin fixed and paraffin embedded minor salivary gland tissue sections were further screened for the presence of germinal center (GC) like structures. Individuals that were evaluated for pSS, but did not fulfill the AECC criteria served as non-pSS tissue controls and were divided into chronically inflamed (CIG) (small focal infiltrates with less than 50 cells; chronically inflamed tissue not sufficient for SS) and normal glands (NG) (no inflammation in the salivary gland tissue). Medical records were obtained from patients' charts at the Department of Rheumatology, Haukeland University Hospital and information was collected regarding routine laboratory assessments including RF detection, antinuclear antibodies (ANA), anti-Ro/SSA, anti-La/SSB, and serum immunoglobulin levels (IgG, IgA and IgM). The characteristics of patients and subjects included in the study are shown in Table 1. The study was approved by the Committee of Ethics at the University of Bergen (145/96-44.96 and 242.06). All studied subjects gave their informed consent.

**Table 1:** Information on patients and subjects included in the study

	Patient/Subject Nr.	Focus score	GC+/-	Age	Gender	Salivary secretion	Ro/SSA	La/SSB	ANA	RF	IgG	IgA	IgM
pSS	1	1	-	48	F	2.0	-	-	0.36	<16	10.0	2.3	1.3
pSS	2	1	-	50	F	1.6	nt	nt	nt	nt	nt	nt	nt
pSS	3	1	+	72	F	1.9	nt	nt	nt	nt	nt	nt	nt
pSS	4	1	-	50	F	1.1	-	-	0.57	<11	10.1	1.2	0.9
pSS	5	1	-	48	F	0.2	-	-	0.68	<11	10.7	0.8	0.9
pSS	6	1	+	62	F	1.7	+	-	2.20	<11	13.9	1.8	3.0
pSS	7	1	-	48	F	0.7	+	-	6.91	nt	19.4	4.1	0.5
pSS	8	1	-	37	F	0.5	-	-	4.55	<11	15.9	3.1	1.7
pSS	9	1	-	68	F	0.8	-	-	2.00	<16	9.8	2.2	0.6
pSS	10	4	-	54	F	0.1	+	-	4.77	32	15.1	4.4	0.9
pSS	11	2	-	58	F	2.5	-	-	0.60	nt**	12.4	1.5	1.3
pSS	12	3	+	40	F	0.2	+	+	nt	nt	nt	nt	nt
pSS	13	4	+	52	F	1.0	-	-	0.17	nt**	12.4	2.7	1.1
pSS	14	12	+	46	F	0	-	-	0.66	nt**	11.9	2.4	1.7
pSS	15	4	-	72	M	0.2	+	+	6.05	nt	16.6	3.7	0.9
pSS	16	4	+	60	F	0.1	-	-	8.05	<11	8.0	1.4	2.1
pSS	17	4	-	56	F	1.2	-	-	0.29	<11	7.1	0.9	1.5
pSS	18	2	-	56	F	0.5	-	-	-*	<11	9.1	0.7	1.8
pSS	19	2	-	50	F	0.8	+	+	1.27	nt**	nt	nt	nt
pSS	20	4	-	58	M	1.5	+	+	9.72	<16	22.1	1.7	0.9
pSS	21	6	+	59	F	4.6	+	+	+*	224**	19.8	1.5	1.4
CIG	22	0	-	46	F	0.9	-	-	0.39	<11	9.0	2.0	0.7
CIG	23	0	-	66	F	4.5	+	-	0.77	<11	19.2	6.4	0.5
CIG	24	0	-	49	F	0.8	-	-	0.1	<11	9.9	1.8	1.0
CIG	25	0	-	70	F	0.9	-	-	0.39	nt	nt	nt	nt
CIG	26	0	-	54	F	1.0	-	-	0.12	<11	14.1	2.0	0.5
NG	27	0	-	54	F	0.2	-	-	0.28	<11	8.8	1.1	0.4
NG	28	0	-	58	F	1.0	-	-	0.45	<11	9.6	1.6	0.8
NG	29	0	-	54	F	2.1	-	-	0.41	nt	nt	nt	nt
NG	30	0	-	55	F	0	nt	nt	nt	nt	nt	nt	nt

CIG, chronically inflamed glands; NG, normal glands; pSS, primary Sjögren's syndrome. Focus score: the number of focal infiltrates of more than 50 mononuclear cells per 4 mm^2^. GC+/-: presence or absence of germinal center like structures based on morphology of the infiltrates. Age, age at the time of SS diagnosis. Gender, Female/Male. Saliva secretion, Mean un-stimulated whole saliva secretion per 15 minutes, normal values ≤1.5 ml/15 minutes. Ro-SSA and La-SSB autoantibodies in serum, positive or negative. ANA, Antinuclear antibodies in serum: negative < 0.99, grey zone 1.00 to 1.65 and positive >1.65. * From 2009 measure as positive or negative. RF, rheumatoid factor in serum: <11 or <16 negative, 32 uncertain. ** Patients with highly positive cyclic citrullinated peptide antibody (CCP). Immunoglobulin levels in serum, normal values: IgG 6.0 to 15.3 g/L, IgA 1.0 to 4.1 g/L and IgM 0.5 to 2.5 g/L. nt, not tested.

### Primary antibodies

The following primary anti-human antibodies were used in this study: mouse monoclonal CD138 (1:00) (clone MI15, Dako A/S, Glostrup, Denmark), rabbit polyclonal IgA (1:50000) (Dako A/S), rabbit polyclonal IgG (1:30000) (Dako A/S), mouse monoclonal Ki-67 (1:150) (clone MIB-1, Dako A/S), rabbit monoclonal Bcl-2 (1:100) (clone E17, Abcam plc., Cambridge, UK), mouse monoclonal CXCL12 (1:250) (clone 79014.111, R&D Systems, Minneapolis, MN USA), rabbit polyclonal IL-6 (1:500) (Abcam plc) and rat anti-PNAd carbohydrated epitope (clone MECA-79, BD Bioscience, Trondheim, Norway). Isotype and concentration matched antibodies (mouse IgG1 and rabbit Ig fraction, Dako A/S) were used as negative controls.

### Immunohistochemistry

#### Single-staining

Formalin fixed, paraffin embedded minor salivary glands were cut (4 to 6 μm) on a Leica serial microtome (Leica Instruments GmbH, Nussloch, Germany) and placed on SuperFrost^® ^Plus microscope slides (Thermo Scientific, Walldorf, Germany). Sections were deparaffinised in xylene and rehydrated through graded ethanol series and distilled water. After heat-induced epitope retrieval (HIER) with citrate buffer (Dako Target Retrieval Solution, pH 6.0, S1699), endogenous peroxidase activity was blocked with 0.03% Dako Peroxidase Block for five minutes. Sections were further incubated with primary antibody (CD138, Bcl-2, IgA, IgG, IL-6 or PNAd) for 60 minutes, followed by incubation with horse radish peroxidase (HRP)-conjugated anti-mouse or anti-rabbit EnVision+ (Dako) for 30 minutes. Except for PNAd, diaminobenzidine (DAB) was used as chromogen for 10 minutes in development of all antibodies. Alkaline phosphatase (AP) and Liquid Permanent Red (LPR, K0640, Dako, Glostrup, Denmark). were used in the development of PNAd staining. All incubations were performed at room temperature (RT) and Tris-buffered saline (TBS) (pH 7.6 with 0.1% Tween) was used for 10 minutes between every step. Sections were counterstained with Haematoxylin (Dako S3301) for three minutes, dehydrated and mounted in Eukitt (O. Kindler GmbH & Co, Freiburg, Germany).

#### Double-staining

Expression of Ki-67/CXCL12/IL-6 and CD138 was detected by double-staining. Sections were pre-treated as described above and Dako Dual Block was used for 10 minutes to block endogenous peroxidase and endogenous AP. The first primary antibody was incubated for one hour at RT followed by the procedure described in single staining. After development of the first primary antibody with DAB, sections were washed with water for 5 minutes, with TBS for 10 minutes and treated with Doublestain Block (Dako) for 3 minutes. Second primary antibody was incubated first for 60 minutes at RT and than over night at 4°C. Binding of the second primary antibody was detected by AP polymer and LPR for seven minutes. Between every step sections were washed with TBS for 10 minutes. Sections were counterstained with Hematoxylin, dehydrated and mounted in Eukitt.

#### Triple-staining

Expression of CD138, PNAd and CXCL12 was detected by triple-staining. Sections were pre-treated as described above and incubated with Dako Dual Block for 10 minutes to block endogenous peroxidase and endogenous alkaline phosphatase. Avidin was used for 15 minutes, TBS for 15 minutes and Biotin for 15 minutes (Blocking Kit Avidin/Biotin, Vector Laboratories, Burlingame, CA, USA) to block endogenous biotin. CD138 was incubated as the first primary antibody. The procedure for single staining was followed. Second primary antibody PNAd was incubated over night at 4°C, followed by 30 minutes incubation with biotinylated rabbit -anti-rat (Dako, E0468) antibody diluted 1:200 in 3% bovine serum albumin (BSA). Development of PNAd was performed by using Vector ABC Kit for 30 minutes and LPR for 7 minutes. The third primary antibody CXCL12 was incubated over night at 4°C followed by incubation with Rabbit/Mouse Link (Dako, K5361) for 30 minutes. Secondary antibody, conjugated with alkaline phosphatase labelled polymer (EnVision+ Dako, Glostrup, Denmark) was applied for 30 minutes. Blue Vector (Vector Laboratories, SK-5300) was used in development of CXCL12 for four minutes. Between every step 10 minutes rinsing with TBS was used. Sections were counterstained with Dako Hematoxylin for one second and mounted in Eukitt.

#### Immunofluorescence staining

Immunofluorescence was used in detection of Bcl-2 expressing plasma cells Sections were pre-treated as described above and non-specific binding was inhibited with TBS buffer containing 2% BSA, 10% normal serum and 0.5% TritonX. Sections were further incubated for one hour at RT with primary antibodies (CD138 and Bcl-2) followed by incubation for two hours with secondary antibody; AlexaFluor488 (green) (Molecular Probes, Invitrogen, Paisley UK) for CD138 and AlexaFluor 594 (red) (Molecular Probes, Invitrogen, Paisley UK) for Bcl-2. Between every step sections were washed with TBS for 10 minutes. Sections were counterstained with DAPI and mounted with Moviol.

#### Evaluation of staining

The minor salivary gland tissue sections were evaluated using a light microscope (Leica DMLB, Leica Microsystems Wetzlar, Wetzlar, Germany) by two investigators. Both interstitial (small mononuclear cell clusters in close proximity to the acinar or ductal epithelium) and mononuclear cells in focal infiltrates were analysed.

Cells were counted using a grid and a 10× or a 40× objective. Cells were considered positive if 50% or more of the cell membrane, nucleus or cytoplasm were positively stained. Intensity of staining was not evaluated in this study. For the analysis of double staining, cells were considered in contact when more than 10% of the cell membranes from both cells were in contact with each other. Counted areas were randomly selected and between three and four minor salivary glands were analysed for each patient or subject. Except for CD138 expression, the data are presented as the number of positive cells/mm^2 ^of salivary gland tissue. For the CD138 expression in the focal infiltrates the data are presented as the percentage of CD138 positive cells of the total number of cells.

### Statistical analysis

Statistical analyses were performed using SPSS version 15.0 software (IBM, Chicago, IL, USA). Normal distributions of the results were tested by Kolmogorov-Smirnov. All data were normally distributed and thus statistic significance was tested by Student *t*-test and presented as mean ± standard error of mean (SEM). Differences were considered significant when *P *< 0.05.

## Results

### Study group

The pSS patients used in this study were divided into two groups according to FS; one group consisted of patients with FS = 1 and one group consisted of patients with FS ≥2. By morphology, we detected GC-like structures (GC+) in salivary glands of 7/21 (33%) patients with pSS. The majority of the GC+ patients were in the FS ≥2 group; mean FS in the GC+ was 4.4 as compared to 2 in the GC- patients (Table 1). Subjects not fulfilling the criteria of pSS were also divided into two groups; 1) subjects with chronic inflammation which presented as diffuse non-focal infiltration of cellular aggregates with less than 50 cells in the minor salivary gland tissue and 2) subjects with normal salivary gland histology; occasional cellular aggregates but not more than expected in normal tissue, and absence of focal inflammation. The mean unstimulated salivary secretion levels were lower than 1.5 ml/15 minutes in all study groups (Table 1). Eight pSS patients were positive for Ro/SSA or/and La/SSB. The mean antinuclear antibodies (ANA) levels in serum were 2.47 in pSS with FS = 1, 3.56 in pSS with FS ≥2, 0.35 in subjects with chronically inflamed glands and 0.77 for subjects with normal gland histology. Only one pSS patient had high amounts of rheumatoid factor in serum; however, five pSS patients were highly positive for cyclic citrullinated peptide antibody (CCP). There were no significant differences in IgG and IgA levels between the groups. The IgM levels were significantly higher in pSS with FS ≥2 compared to chronically inflamed (*P *< 0.05) and normal (*P *< 0.05) subjects (Table 1).

### High number of CD138 expressing plasma cells detected in pSS with FS ≥2

Plasma cells in the salivary gland tissue identified by the expression of CD138 showed both plasma blast and mature plasma cell morphology. Cells with mature plasma cell morphology had characteristic eccentrically placed nucleus, prominent cytoplasm and oval shape, whereas cells with plasma blast morphology were smaller, spherical and with higher nucleus-to-cytoplasm ratio.

**Figure 1:**
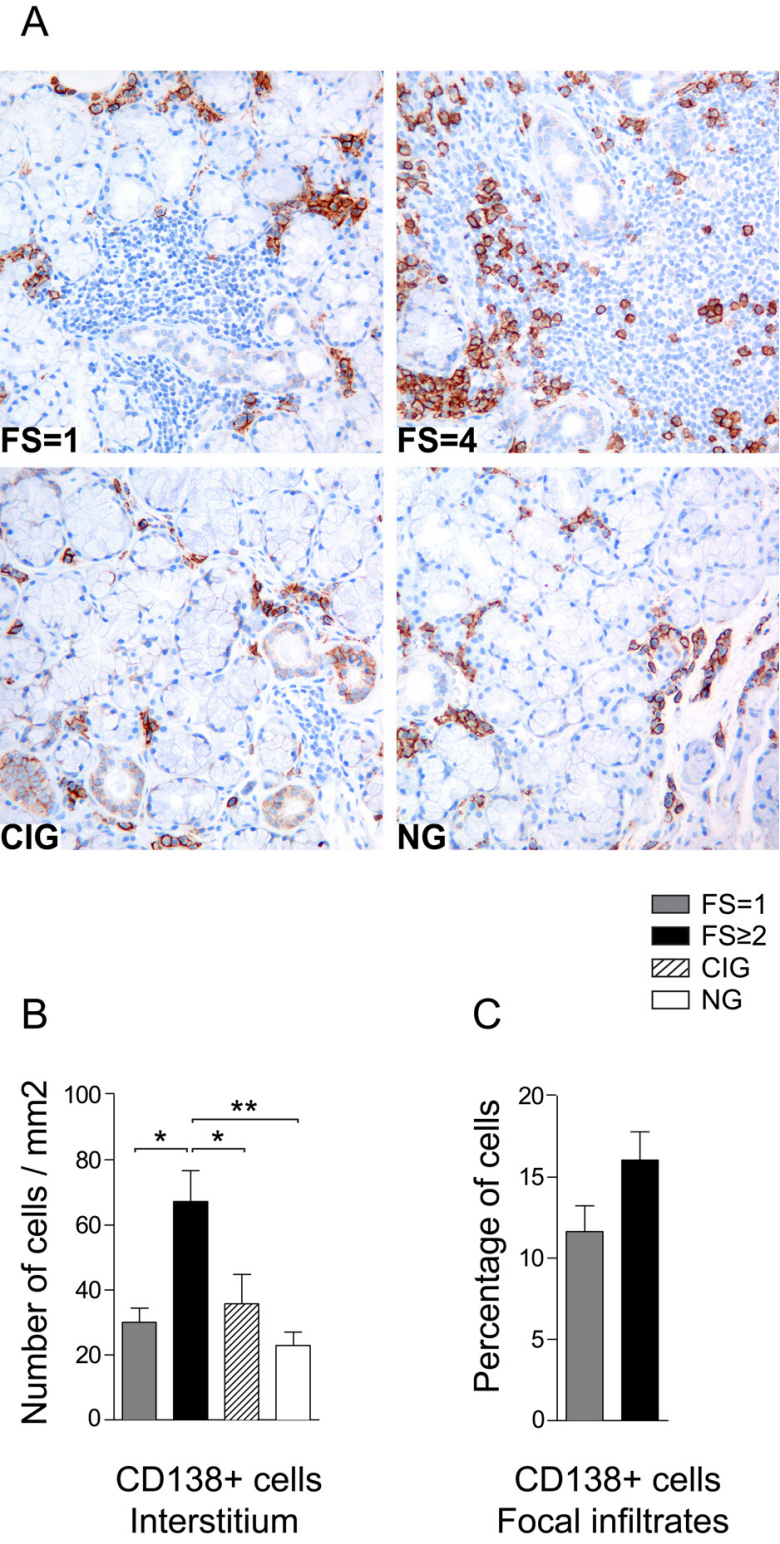
**CD138 expressing plasma cells in salivary glands of pSS patients and non-SS controls**. **A) **CD138+ plasma cells (brown) in pSS patient with FS = 1, a pSS patient with FS = 4, in a subject with chronically inflamed glands (CIG) and a subject with normal gland (NG) histology. CD138+ plasma cells were detected interstitially as small cell clusters close to ductal and acinar epithelium in all patients and controls. In pSS glands with FS = 1, plasma cells were detected mainly in the periphery of the infiltrates, whereas in pSS glands with high FS, plasma cells were detected both in periphery and within the infiltrates. **B) **CD138+ cells presented as number of interstitial plasma cell per mm^2 ^or as **C) **percentage of CD138 positive plasma cells in the focal infiltrates. Grey bars represent FS = 1 (*n *= 6), black bars represent FS ≥2 (*n *= 10), striped bars represent chronic inflammation (*n *= 5) and white bars represent normal gland histology (*n *= 5). Results presented as mean ± SEM. Statistical analyses determined by Kolmogorov-Smirnov and Student *t-*test (*P < 0.05, **P < 0.005).

CD138+ plasma cells were detected in the salivary gland tissue of all investigated patients. In patients with FS = 1, CD138 expressing cells were detected interstitially as small cell clusters close to ductal and acinar epithelium and in the periphery of larger infiltrates. In the biopsy samples from patients with FS ≥2, CD138 expression was identified interstitially and both in the periphery and within larger focal infiltrates (Figure 1A).

Further, we detected CD138+ plasma cells within some GC-like structures as described in more detail later.

Significantly higher numbers of CD138+ interstitial cells were detected in pSS patients with FS ≥2 compared to the other groups tested (Figure 1B). There were no significant differences in the amount of CD138+ interstitial cells between the pSS group with FS = 1, chronic inflammation and normal glands (Figure 1B). When looking at focal infiltrates, we found no significant differences between the percentage of CD138+ cells in pSS patients with FS = 1 and pSS patients with FS ≥2 (Figure 1C). In our material, the presence of GC-like structures had no impact on the numbers of detected CD138 plasma cells.

### Up-regulation of IgG but not IgA expressing plasma cells detected in pSS with FS ≥2

**Figure 2:**
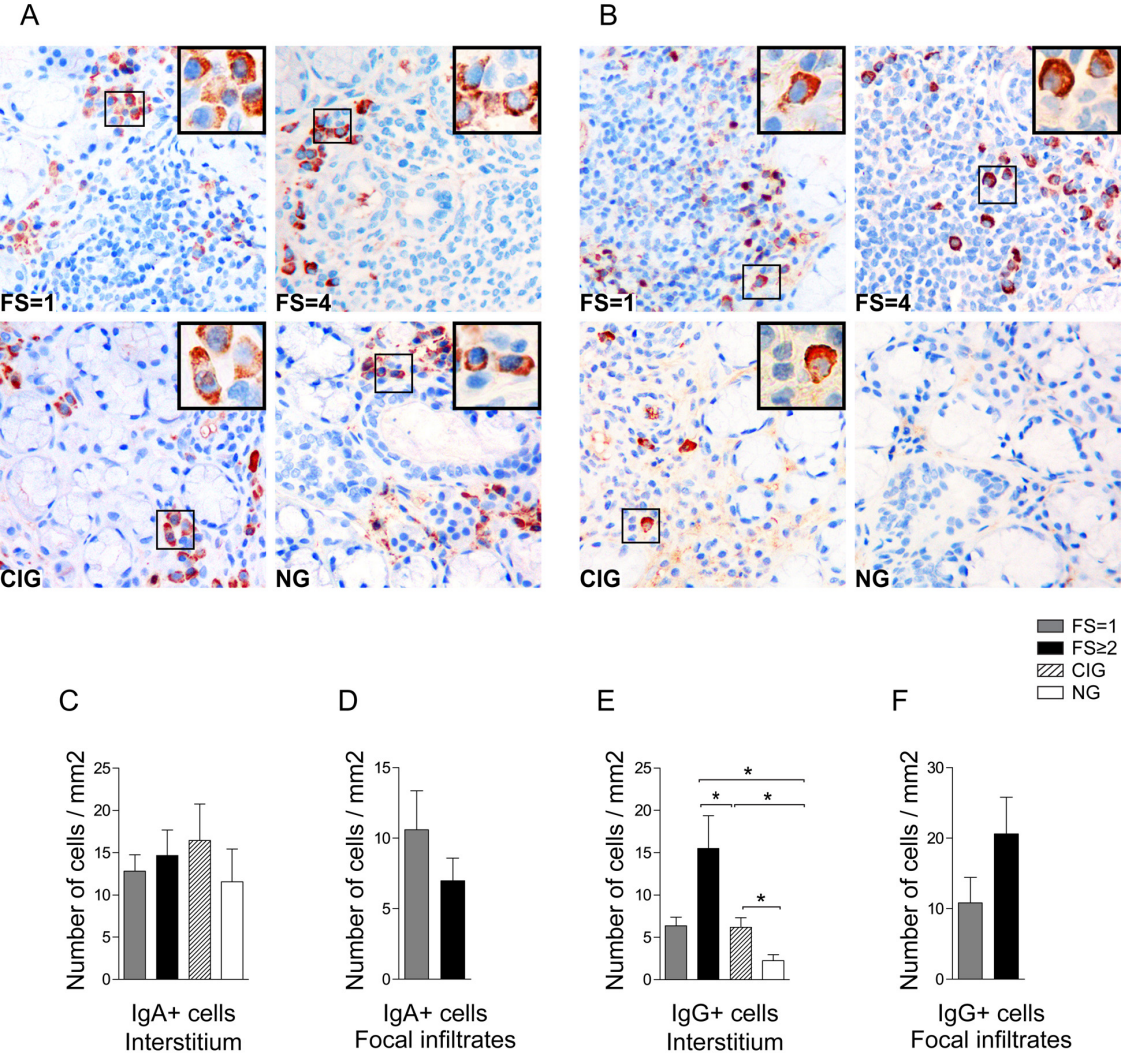
**IgA and IgG expressing plasma cells in salivary glands of pSS patients and non-SS controls**. **A) **IgA and **B) **IgG positive plasma cells detected in salivary glands of a pSS patient with FS = 1, a pSS patient with FS = 4, in a subject with chronically inflamed glands (CIG) and a subject with normal gland (NG) histology. Inserts represent higher magnifications of IgA or IgG plasma cells for each group. IgA expressing plasma cells were detected interstitially as small cell clusters close to ductal and acinar epithelium in all groups tested and in the periphery of infiltrates in pSS with FS = 1 and FS ≥2 (A). IgG expressing plasma cells were found both inside and in the periphery of large focal infiltrates in pSS patients with FS = 1 and FS ≥2, whereas interstitial accumulation of IgG expressing plasma cells was observed mostly in the pSS patients with high FS (B). **C) **Amount of IgA or **E) **IgG positive interstitial plasma cells presented as number of cells/mm^2 ^and **D) **IgA or **F)** IgG positive cells in the focal infiltrates presented as number of positive cells per mm^2^. Grey bars represent FS = 1 (*n *= 8), black bars represent FS ≥2 (*n *= 7), striped bars represent chronic inflammation (*n *= 5) and white bars represent normal gland histology (*n *= 5). Results presented as mean ± SEM. Statistical analyses determined by Kolmogorov-Smirnov and Student *t *test (*P < 0.05).

An analysis of immunoglobulin phenotype showed the presence of both IgA and IgG expressing plasma cells in the salivary gland tissue of all patients and subjects tested (Figure 2). IgA expressing plasma cells were detected in small cell clusters in close proximity to ductal and acinar epithelium in all groups tested and in the periphery of smaller and larger focal infiltrates in pSS salivary glands. IgA+ cells clearly resembled plasma cell morphology (Figure 2A). IgG expressing plasma cells were found both inside and in the periphery of large focal infiltrates in pSS patients with FS = 1 and FS ≥2, whereas interstitial accumulation of IgG expressing plasma cells was observed mostly in the pSS patients with high FS. Similarly to IgA, IgG+ cells clearly resembled plasma cell morphology (Figure 2B).

Approximately the same frequencies of IgA expressing plasma cells were noted in the interstitial areas of all three groups tested (Figure 2C). However, not significantly, a reduction in IgA+ plasma cells was noted in the focal infiltrates of pSS patients with FS ≥2 (Figure 2D).

Both pSS groups (FS = 1 and FS ≥2) and subjects with chronically inflamed salivary glands had significantly higher numbers of interstitial IgG plasma cells compared with normal tissue. However, pSS patients with high FS demonstrated the most prominent increase in the interstitial IgG plasma cells (Figure 2E). The number of IgG expressing plasma cells in the focal infiltrates was higher in the pSS with FS ≥2 but due to great variation within the groups, the differences were not significant (Figure 2F).

Interestingly, the focal infiltrates of pSS patients with FS = 1 contained similar frequencies of IgA and IgG expressing plasma cells, whereas in the focal infiltrates of pSS patients with high FS, the numbers of IgG expressing plasma cells were three times higher than numbers of IgA positive plasma cells (Figure 2D, F). The presence of GC-like structures appeared to have no impact on the amounts of IgA or IgG expressing plasma cells. No correlation was detected when we compared the increased number of IgG versus IgA plasma cells in the salivary glands and serum IgG and IgA levels in patients with pSS.

### Accumulation of non-proliferative plasma cells in salivary gland tissue

Since it has been proposed that the long-lived plasma cells survive without detectable proliferation [21,32], a Ki-67/CD138 double-staining was performed.

**Figure 3:**
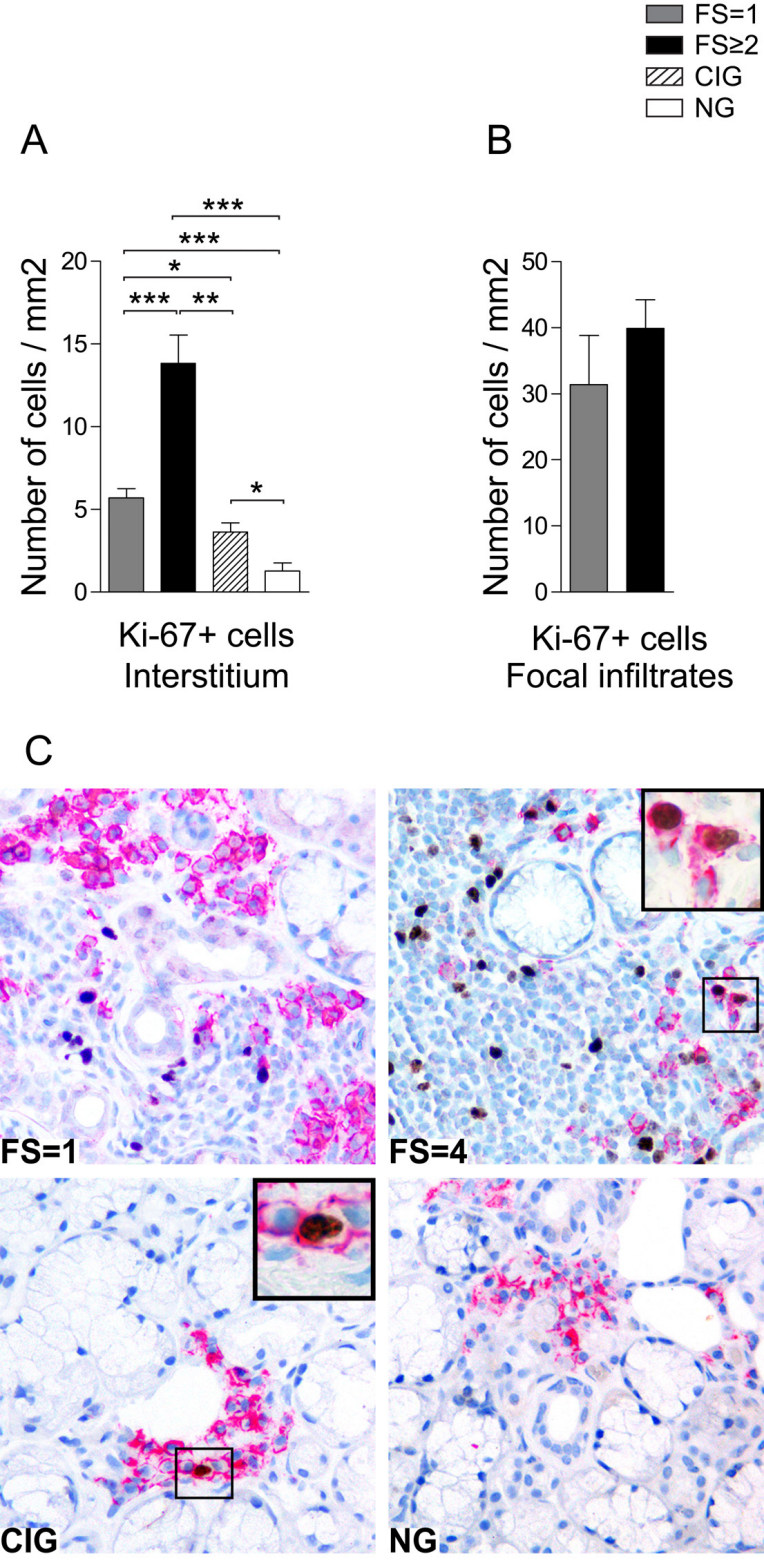
**Proliferating and non-proliferating plasma cells in salivary glands of pSS patients and non-SS controls**. **A) **Amount of Ki-67+ interstitial cells and **B) **Ki-67+ cells in the focal infiltrates presented as number of cells per mm^2^. Grey bars represent FS = 1 (*n *= 8), black bars represents FS ≥2 (*n *= 7), striped bars represent chronic inflammation (*n *= 5) and white bars represent normal gland histology (*n *= 5). Results presented as mean ± SEM. Statistical analyses determined by Kolmogorov-Smirnov and Student *t *test (*P < 0.05, **P < 0.005 and ***P 0.001). **C) **Double-staining with CD138 plasma cell marker (red) and Ki-67 proliferation marker (brown) in a pSS patient with FS = 1, in a pSS patient with FS = 4, in a subject with chronically inflamed glands (CIG) and a subject with normal gland (NG) histology. Inserts represent higher magnifications of CD138+ Ki-67+ plasma cells. Ki-67+ plasma cells were mainly observed in the periphery of larger infiltrates with high proliferating activity and in some small plasma cell aggregates often in close proximity to vascular structures.

Ki-67 was generally expressed on mononuclear cells in the focal infiltrates of pSS patients with both FS = 1 and FS ≥2 and in the interstitial cells of all groups included in this study (Figure 3). Frequencies of interstitially Ki-67+ cells were low in the normal tissue, but increased significantly in chronically inflamed and in pSS tissue with FS = 1. A remarkable increase of Ki-67+ interstitial cells was observed in the salivary glands of pSS patients with FS ≥2 (Figure 3A). Only a slight difference was detected in the expression of Ki-67 by focally infiltrating cells in pSS patients with FS = 1 and in pSS patients with FS ≥2 (Figure 3B). Moreover, GC positivity did not seem to associate with the numbers of focally infiltrating Ki-67+ cells.

Most of the CD138+ plasma cells did not express Ki-67. For the pSS groups with FS = 1 and FS ≥2 we detected on average four double-positive cells per gland. For the group with chronic inflammation we detected one double-positive cell per gland, whereas no double positive cells were detected in normal salivary gland tissue. Ki-67+ plasma cells were mainly observed in the periphery of larger infiltrates with high proliferating activity and in some small plasma cell aggregates often in close proximity to vascular structures (Figure 3C).

### Bcl-2 expressing plasma cells preferentially associate with pSS high FS

In order to determine whether the accumulating plasma cells in the minor salivary glands of pSS patients and controls are primed to survive for long periods of time [33,34] we looked at the expression of anti-apoptotic Bcl-2 protein.

**Figure 4:**
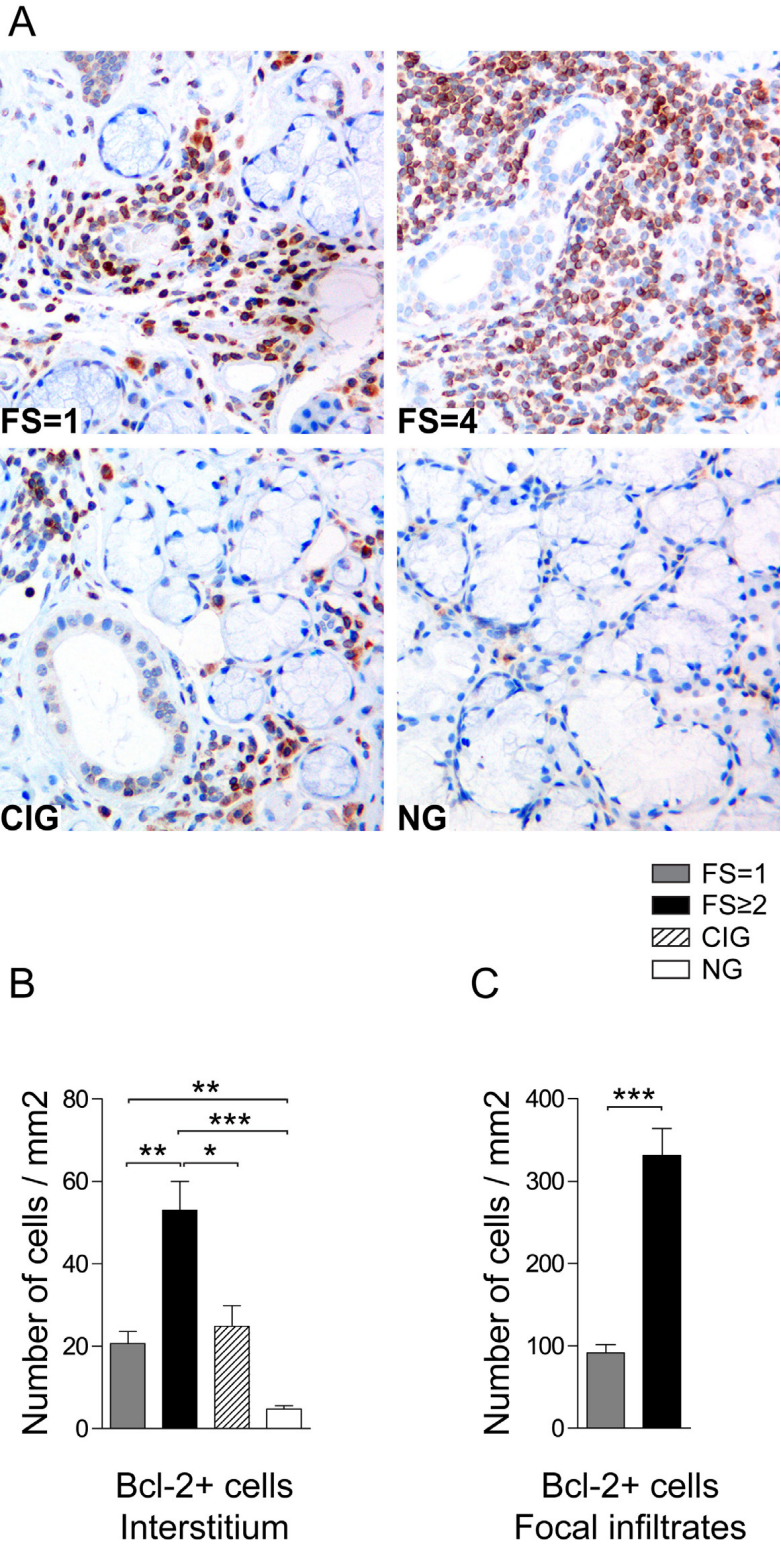
**Bcl-2 expression in infiltrating lymphocytes of pSS patients and non-SS controls**. **A) **Bcl-2 detected in salivary glands of a pSS patient with FS = 1, a pSS patient with FS = 4, in a subject with chronically inflamed glands (CIG) and a subject with normal gland (NG) histology. Bcl-2 was expressed in the cytoplasm of mononuclear cells both in the focal infiltrates of pSS patients and interstitially in all study groups. **B) **Amount of Bcl-2+ interstitial cells and **C) **Bcl-2+ cells in the focal infiltrates presented as number of cells per mm^2^. Grey bars represent FS = 1 (*n *= 8), black bars represent FS ≥2 (*n *= 7), striped bars represent chronic inflammation (*n *= 5) and white bars represent normal gland histology (*n *= 5). Results presented as mean ± SEM. Statistical analyses determined by Kolmogorov-Smirnov and Student *t *test (*P < 0.05, **P < 0.005 and ***P < 0.001).

Bcl-2 was expressed in the cytoplasm of mononuclear cells both in the focal infiltrates of pSS patients and interstitially in all study groups (Figure 4A). We observed significantly higher expression of Bcl-2 in the interstitial cells of pSS patients with FS ≥2 compared with the three other groups (Figure 4B). Furthermore, significantly higher numbers of Bcl-2+ interstitial cells were found in the pSS tissue with FS = 1 comparing with normal, but not with chronically inflamed tissue (Figure 4B). When evaluating focal infiltrates, a dramatic increase in the levels of Bcl-2 expressing cells was observed in the pSS with high FS comparing with pSS with FS = 1 (Figure 4C). GC positivity did not seem to influence the numbers of Bcl-2 positive cells in the salivary gland tissue included in this study.

**Figure 5:**
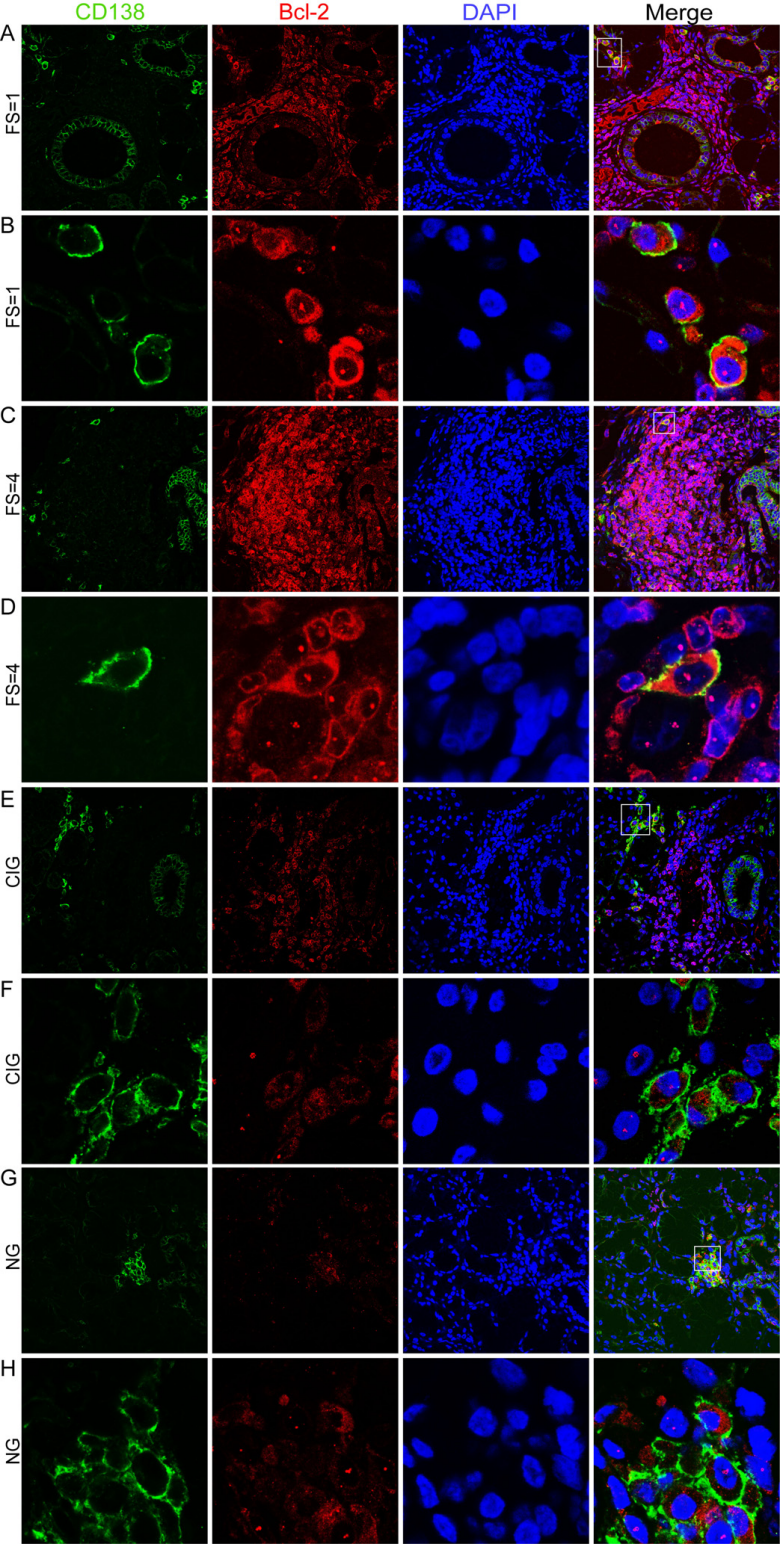
**Bcl-2 expression in infiltrating plasma cells of pSS patients and non-SS controls**. **A) **CD138 (green) and Bcl-2 expressing cells (red) detected in a pSS patient with FS = 1. DAPI (blue) was used for nuclear staining of salivary glands cells. Merge image shows double positive (CD138 and Bcl-2) cells. **B) **Higher magnifications of CD138 (green) and Bcl-2 (red) expressing cells in a pSS patient with FS = 1. Merge image with CD138+ Bcl-2+ plasma cells. Only Bcl-2+ plasma cells were detected in the salivary glands of pSS patients with FS = 1. **C) **CD138 (green) and Bcl-2 expressing cells (red) detected in a pSS patient with FS = 4. DAPI (blue) was used for nuclear staining of salivary glands cells. **D) **Higher magnifications of CD138 (green) and Bcl-2 (red) expressing cells in a pSS patient with FS = 4. Merge image with CD138+ Bcl-2+ plasma cell. Only Bcl-2+ plasma cells were detected in the salivary glands of pSS patients with FS ≥2. **E) **CD138 (green) and Bcl-2 expressing cells (red) detected in a subject with chronically inflamed glands (CIG). DAPI (blue) was used for nuclear staining of salivary glands cells. **F) **Higher magnifications of CD138 (green) and Bcl-2 (red) expressing cells in a subject with CIG. Merge image with CD138+ Bcl-2+ and CD138+ Bcl-2- plasma cells. Both Bcl-2+ and Bcl-2- plasma cells were detected in the chronically inflamed tissue. **G) **CD138 (green) and Bcl-2 expressing cells (red) detected in a subject with normal gland (NG) histology. DAPI (blue) was used for nuclear staining of salivary glands cells. **H) **Higher magnifications of CD138 (green) and Bcl-2 (red) expressing cells in a subject with NG histology. Merge image with CD138+ Bcl-2+ and CD138+ Bcl-2- plasma cells. Both Bcl-2+ and Bcl-2- plasma cells were detected in the normal tissue.

Notably, no Bcl-2 negative plasma cells could be detected in the salivary glands of pSS patients (both FS = 1 and FS ≥2) (Figure 5A-D). In the chronically inflamed and normal tissue both Bcl-2+ and Bcl-2- plasma cells were observed (Figure 5E-H)). Even though it is difficult to evaluate the intensity of the Bcl-2 expression, a more distinct cytoplasmic Bcl-2 staining was noted in plasma cells from pSS salivary glands compared to plasma cells from subjects with chronic inflammation and normal salivary gland tissue (Figure 5).

### Plasma cells in pSS salivary glands located in close contact with CXCL12 expressing cells

CXCL12 (also known as stromal derived factor 1; SDF-1) plays a crucial role in the migration and survival of plasma cells [22,23,27]. To investigate the possibility of CXCL12 to facilitate plasma cell survival in the inflamed salivary gland tissue, a double-staining with anti-CXCL12 and anti-CD138 was performed.

**Figure 6:**
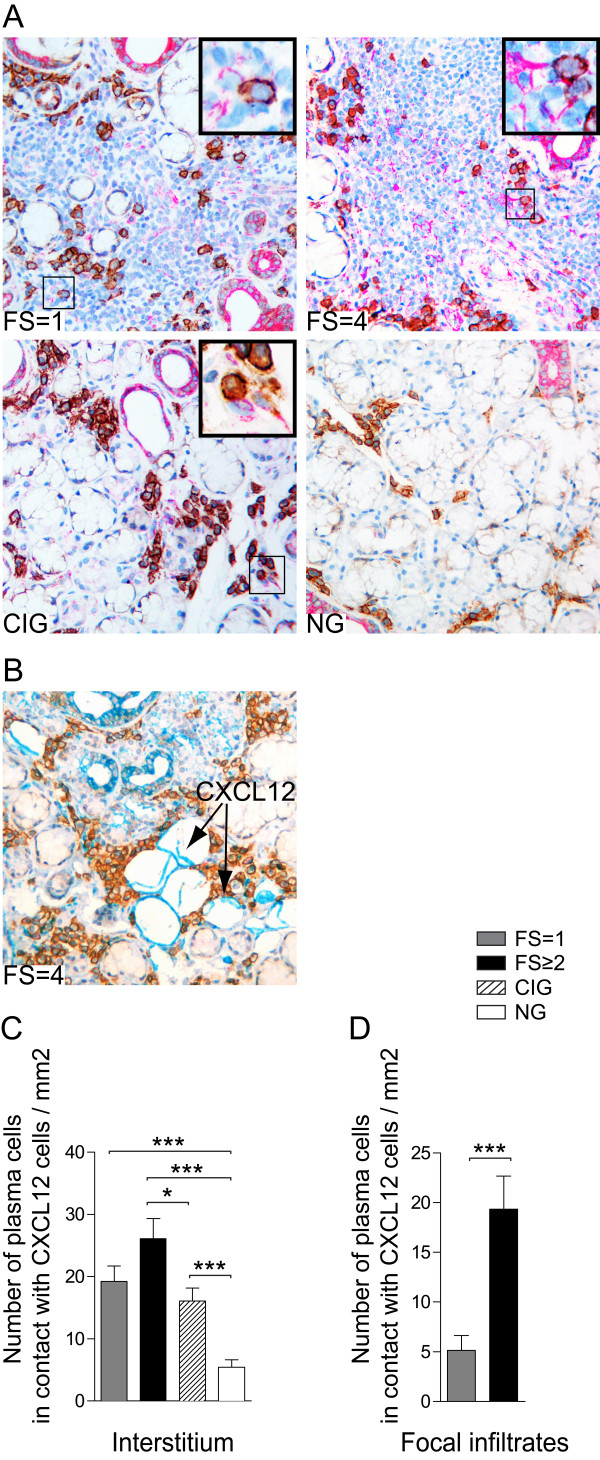
**Double staining of CXCL12 and CD138 in salivary glands of pSS patients and non-SS controls**. **A) **Double-staining with CD138 plasma cell marker (brown and CXCL12 (red) in a pSS patient with FS = 1, a pSS patient with FS = 4, in a subject with chronic inflammation and a subject with normal gland histology. Inserts represent higher magnifications of CD138+ plasma cells (brown) in contact with CXCL12 expressing cells (red). CXCL12 was detected on acinar and ductal epithelium in all three groups tested and on the mononuclear cells in pSS and chronically inflamed but not normal salivary glands. Plasma cells were in contact with both CXCL12 expressing epithelium and CXCL12 expressing cells. **B) **Expression of CXCL12 (blue) on adipocytes in a pSS patient with FS = 4. Plasma cells were in close contact with CXCL12 expressing adipocytes in pSS glands with high FS. **C) **Amount of CD138+ interstitial plasma cells in contact with CXCL12+ cells and **D) **Amount of CD138 positive cells in contact with CXCL12 positive cells in the focal infiltrates presented as number of cells per mm^2^. Grey bars represent FS = 1 (*n *= 6), black bars represent FS ≥2 (*n *= 8), striped bars represent chronic inflammation (*n *= 5) and white bars represent normal gland histology (*n *= 5). Results presented as mean ± SEM. Statistical analyses determined by Kolmogorov-Smirnov and Student *t-*test (*P < 0.05 and ***P < 0.001).

CXCL12 staining was detected on acinar and ductal epithelial cells in pSS patients and to a lesser extent in chronic inflammation and normal subjects (Figure 6A). CXCL12 was also expressed on the mononuclear cells interstitially in pSS and chronic inflammation subjects and in focal infiltrates of all pSS patients. No expression of CXCL12 was detected on the interstitial cells in normal salivary gland tissue (Figure 6A). Additionally, we observed expression of CXCL12 by resident adipocytes present in some areas of salivary gland tissue of pSS patients (Figure 6B).

In pSS patients and chronic inflammation subjects, CD138+ interstitial plasma cells were mainly observed in close proximity to the CXCL12 expressing ductal and acinar epithelium and CXCL12 expressing mononuclear cells (Figure 6A). In contrast, in normal tissue clusters of CD138+ plasma cells were mainly observed in close proximity to CXCL12 negative acini. Interestingly, small aggregates of CD138+ plasma cells were in close contact to CXCL12 expressing adipocytes in pSS salivary gland tissue.

Significantly higher numbers of CD138+ interstitial plasma cells were in contact with CXCL12 expressing cells in pSS patients with FS ≥2 compared to chronic inflammation and normal subjects (Figure 6C). However, there were no statistical differences in the numbers of interstitial plasma cells in contact with CXCL12 between pSS with FS = 1 and chronic inflamed tissue. In normal glands, only few plasma cells were in contact with CXCL12 expressing epithelium (Figure 6C).

Plasma cells residing in the focal infiltrates of pSS patients were in contact with CXCL12 expressing cells (Figure 6A). The frequency of interactions between the CD138+ plasma cells and CXCL12 were significantly increased in the focal infiltrates of pSS with FS ≥2 compared with interactions seen in the pSS tissue with FS = 1 (Figure 6D).

### Plasma cells in pSS salivary glands accumulate in IL-6 rich niches

IL-6 cytokine has proven to be vital for plasma cell survival [22]. Thus, we performed a double-staining for IL-6 and CD138, to investigate if IL-6 can support the survival of plasma cells in the salivary gland tissue.

**Figure 7:**
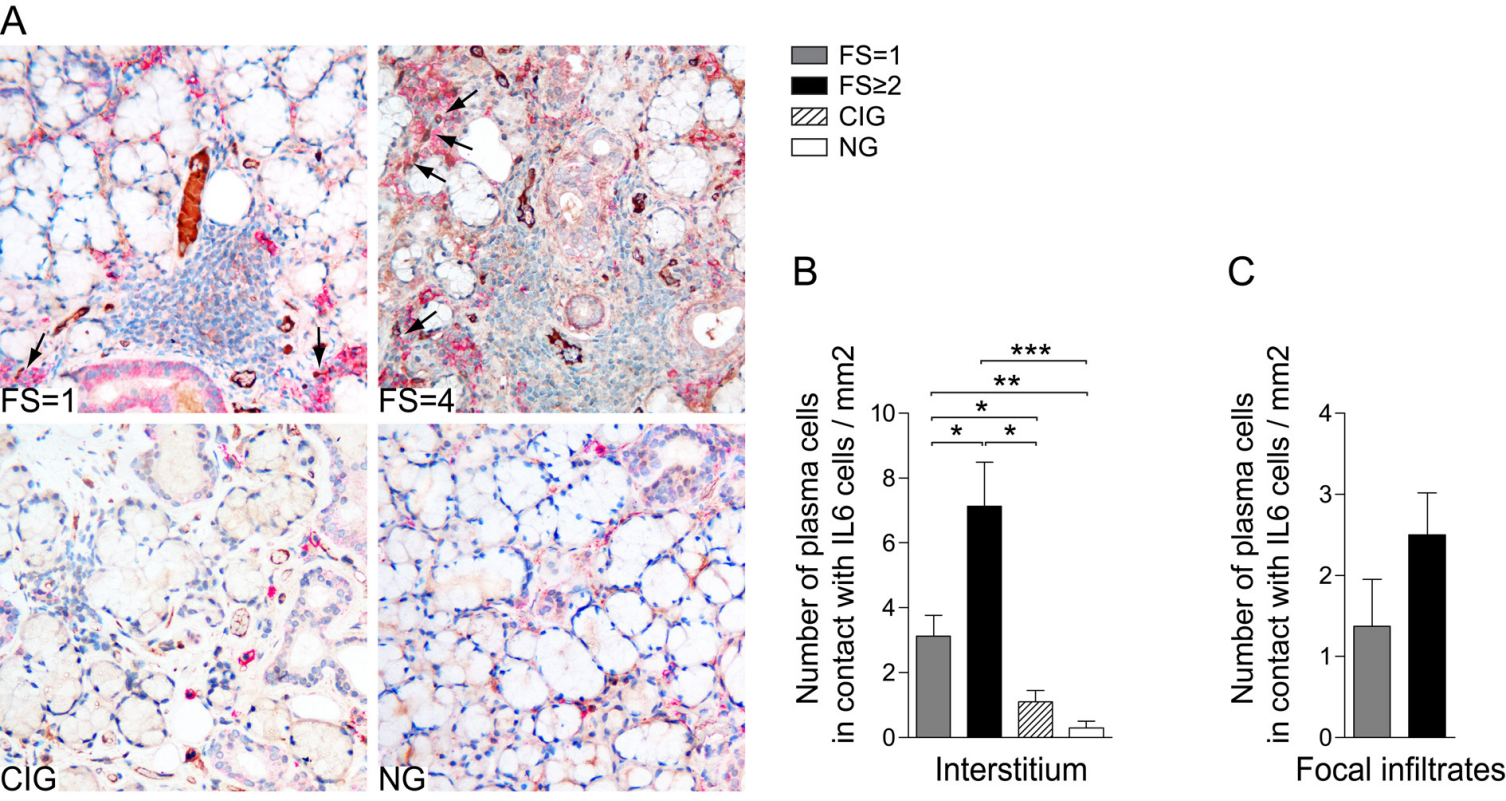
**Double staining of IL-6 and CD138 in salivary glands of pSS patients and non-SS controls**. **A) **IL-6 expression (brown) and CD138+ plasma cells (red) detected in a pSS patient with FS = 1, a pSS patient with FS = 4, in a subject with chronic inflammation and a subject with normal gland histology. IL-6 was expressed on the interstitial cells in all groups studied and the majority of salivary gland epithelial cells were negative for IL-6. In the focal infiltrates IL-6 expressing cells were mostly detected in pSS patients with FS ≥2. Interaction between CD138+ plasma cells and IL-6 expressing cells were detected in the pSS glands with both FS = 1 and FS ≥2 and not in chronically inflamed or normal glands. Interactions between IL-6 and CD138 plasma cells are indicated by arrows. **B) **Amount of CD138+ interstitial cells in contact with IL-6+ cells and **C) **amount of CD138+ plasma cells in contact with IL-6+ cells in the focal infiltrates presented as number of cells per mm^2^. Grey bars represent FS = 1 (*n *= 9), black bars represent FS ≥2 (*n *= 8), striped bars represent chronic inflammation (*n *= 5) and white bars represent normal gland histology (*n *= 5). Results presented as mean ± SEM. Statistical analyses determined by Kolmogorov-Smirnov and Student *t*-test (*P < 0.05, **P < 0.005 and ***P < 0.001).

IL-6 was expressed primarily on the salivary gland interstitial cells in all groups studied and the majority of salivary gland epithelial cells were negative for this cytokine (Figure 7). Only scattered IL-6+ acinar epithelial cells were detected in some of the pSS patients (data not shown). IL-6 was demonstrated within the majority of the focal infiltrates; however, the number of cells expressing IL-6 was generally higher in the pSS patients with FS ≥2 (Figure 7A).

Salivary glands from pSS patients had significantly higher numbers of CD138+ plasma cells in close contact with IL-6 producing cells than plasma cells in chronically inflamed and normal tissue (Figure 7B). Moreover, the number of plasma cells that interacted with IL-6, increased drastically from pSS patients with FS = 1 to pSS with FS ≥2 (Figure 7B). Since only few IL-6 expressing cells were detected in chronically inflamed and normal subjects, the contact between these cells and CD138+ plasma cells was rare (Figure 7B).

When investigating focal infiltrates, we detected higher numbers of CD138+ plasma cells in close proximity to the IL-6 producing cells in the pSS with high FS compared with pSS with FS = 1. However, due to variation in the presence of IL-6 producing cells, these differences were not significant (Figure 7C).

### High endothelial venules retain strongly IL-6 and more moderately CXCL12 in pSS salivary glands

Formation of specific blood vessels called high endothelial venules (HEVs) has been detected in the inflamed tissue of autoimmune diseases such as the synovium in rheumatoid arthritis and in salivary gland tissue of SS patients [35-37]. However, little is known about the phenotype and the function of these structures at the site of inflammation. To further characterize the distribution pattern of HEVs in the salivary glands of pSS and in particular their possible contribution to 'survival niches' for plasma cells, we performed for the first time a IHC triple staining with PNAd, CXCL12 and CD138. In addition, consecutive salivary gland tissue sections were single stained with PNAd and IL-6.

**Figure 8:**
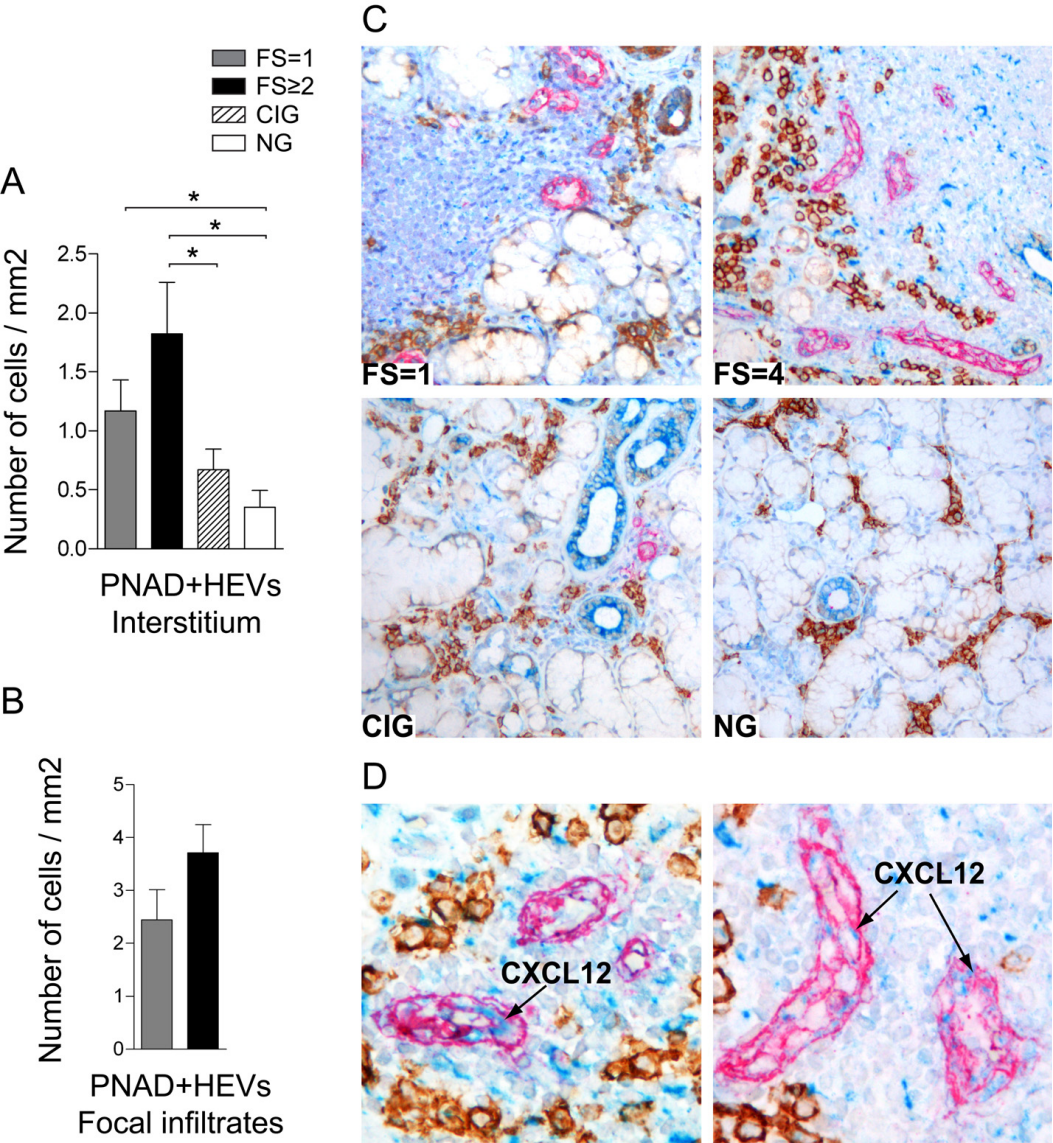
**Triple staining of CXCL12, PNAd and CD138 in salivary glands of pSS patients and non-SS controls**. **A) **Amounts of PNAd+ HEVs in the interstitium and **B) **in the focal infiltrates in salivary glands presented as number of cells per mm^2^. Grey bars represent FS = 1 (*n *= 9), black bars represent FS ≥2 (*n *= 10), striped bars represent chronic inflammation (*n *= 5) and white bars represent normal gland histology (*n *= 5). Results presented as mean ± SEM. Statistical analyses determined by Kolmogorov-Smirnov and Student *t-*test (*P < 0.05, **P < 0.005 and ***P < 0.001). **C) **CXCL12 expression (blue), PNAd expression (red) and CD138+ plasma cells (brown) detected in a pSS patient with FS = 1, a pSS patient with FS = 4, in a subject with chronic inflammation and a subject with normal gland histology. PNAd +HEVs were mainly detected in the close proximity or inside of the infiltrates in the pSS glands. CD138+ plasma cells were observed tightly adherent to both CXCL12 positive and CXCL12 negative HEVs. However, only few HEVs expressed CXCL12. **D) **Higher magnification of CXCL12 expression (blue) detected in some of the PNAd+ HEVs (red).

HEVs were detected interstitially in all groups tested. In pSS patients, HEVs were often localized in close proximity to and within focal infiltrates. Occurrence of HEVs increased with increasing inflammation and thus high numbers were detected in pSS patients with FS ≥2 (Figure 8A, B). Using the triple staining, only few of the HEV endothelial cells in pSS salivary glands were expressing CXCL12. CD138+ plasma cells were often observed in an extravascular location, sometimes tightly adherent to both CXCL12 positive and CXCL12 negative HEVs (Figure 8C, D). No CXCL12 positive HEVs were recognized in chronically inflamed or in normal salivary gland tissue.

**Figure 9:**
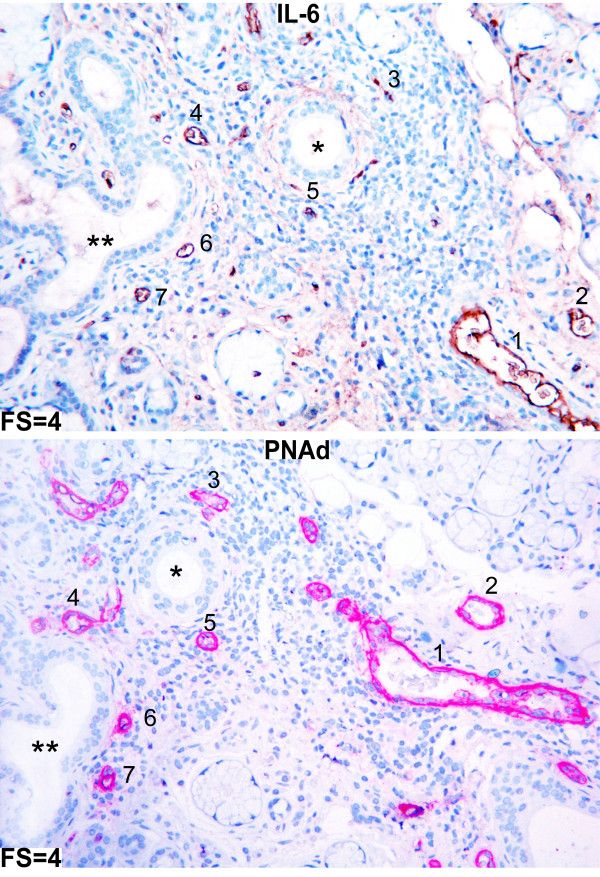
**Serial staining of IL-6 and PNAd in salivary glands of a pSS patient**. IL-6 and PNAd stainings of pSS glands were performed as serial immunohistochemistry to detect possible colocalisation. The same structures in the two sections are marked as * and **. Colocalisations of IL-6 and PNAd are marked with numbers from 1 to 7. Most of HEVs expressed IL-6.

To gain further insight into the mode of chemokine binding to HEVs, we next investigated the possible relationship between IL-6 and HEVs in the salivary glands. Indeed most of HEVs in our selected pSS material expressed IL-6 (Figure 9).

## Discussion

The present study demonstrates that minor salivary glands of pSS patients provide niches rich in specific factors vital for survival of plasma cells. Notably, salivary glands from pSS patients with severe inflammation (high FS) expressed significantly more of these factors than the three other groups tested. To our knowledge, we are the first to demonstrate by using double and triple immunohistochemistry, that CD138+ plasma cells are located in close proximity to ductal and acinar epithelium as well as to mononuclear cells within the focal infiltrate expressing survival factors CXCL12 and IL-6. An association between the accumulation of plasma cells and CXCL12 expressing HEVs were also detected exclusively in pSS salivary gland tissue. Furthermore, plasma cells accumulating in the salivary glands of pSS were found to be non-proliferating, Bcl-2 expressing cells and could possibly be the long-lived plasma cell subset.

When investigating the presence of plasma cells, we detected significantly higher numbers of these cells in the salivary gland tissue of pSS patients with FS ≥2 compared to the three other groups. Interestingly, the numbers of plasma cells in FS = 1 group did not differ from chronically inflamed and normal group. The regulation of plasma cell differentiation and accumulation in the inflamed salivary gland tissue is complex and not defined. The significant increase in plasma cells in the salivary glands with high FS could be due to increased migration into the tissue and/or a consequence of B-cell activation and differentiation in the ectopic germinal centres that can be formed in the salivary glands of SS patients [9,17,38,39]. In correlation with previous studies [17], the presence of GC-like structures was detected mostly in pSS patients with more severe inflammation (high FS). No association was noted between the number of plasma cells and presence of GC-like structures in our patients. However, based on the limited GC+ patients included in this study only a trend could be indicated. Another possibility could be that plasma cells are produced in the tissue without involvement of GC-like structures as it has been shown that class-switch recombination and somatic hypermutation of B-cells can also occur outside of GC [40]. In any case, whether migrated or not, the presence of high numbers of CD138+ plasma cells implies that the microenvironment of the salivary glands with high FS provide factors/signals necessary for the maintenance and survival of these cells.

There has been some interest in the IgG versus IgA plasma cell expression in the salivary glands of SS patients especially between the primary and secondary SS. In agreement with previous IHC study on SS salivary gland tissue [41], examination of immunoglobulin phenotype of plasma cells in our study, revealed significantly higher numbers of interstitial IgG plasma cells in pSS patients compared with non-SS control. Additionally, our study reveals that when divided in groups, pSS patients with FS ≥2 had significantly more interstitial IgG plasma cells but not focally infiltrating cells than pSS patients with FS = 1. Hence, we show that the increase in IgG plasma cells is associated with more severe inflammation in the salivary gland tissue of pSS patients.

In contrast to a previous report [41], we detected similar IgA profiles in all three groups tested. Thus, it seems that the IgG plasma cells are the ones responsible for the increase of the CD138+ plasma cells that we observed in the salivary glands with high FS. However, further studies by use of double staining are needed to confirm this hypothesis. These findings are consistent with our previous studies [42] in which a similar predominance of IgG versus IgA autoantibody production by single cells dispersed from SS salivary glands was detected by use of the ELISPOT essay.

Based on studies of murine bone marrow and splenic plasma cells, it has been speculated whether non-proliferating plasma cells are cells that are able to survive for long period of times [21,32]. In this context, we examined the proliferation and survival capacity of salivary gland plasma cells by using double staining immunohistochemistry (IHC) with CD138/Ki-67 and CD138/Bcl-2. Interestingly, as detected by the lack of Ki-67, plasma cells present in the salivary glands of all three groups tested were non proliferating cells.

In addition, all CD138 plasma cells present in salivary glands of pSS patients expressed high levels of anti-apoptotic Bcl-2 protein, whereas both Bcl-2+ and Bcl-2- plasma cells were present in the chronically inflamed and normal tissue. This points to the fact that plasma cells marked for survival are unregulated in the salivary glands of pSS patients compared to non-SS salivary gland tissue.

Bcl-2 is a protein that regulates survival of lymphocytes in a mitochondria-dependent way and has previously been detected in plasma cells isolated from blood, bone marrow and to some extent in plasma cells from tonsils [33,34]. Its importance in regulation of anti-apoptotic processes in the plasma cell population has been extensively demonstrated in multiple myeloma cells [43-45]. In accordance, the potential of IL-6 to induce Bcl-2 expression has been demonstrated [46]. Whether IL-6 survival factor identified in this study induce the Bcl-2 expression detected in pSS salivary gland plasma cells, remains to be elucidated.

There is increasing evidence suggesting that the life time of plasma cells is determined by the environment rather than intrinsic factors [33]. Bone marrow studies have revealed that a proper plasma cell survival environment comprises chemokines and cytokines such as CXCL12 and IL-6 [22,23,27-29]. Based on these observations, we characterized in detail the expression pattern of CXCL12 and IL-6 in the salivary glands of pSS patients, chronically inflamed and normal salivary gland tissue.

We detected CXCL12 on acinar and ductal epithelium in all study groups; however the most prominent expression of this chemokine was detected in pSS patients with FS ≥2. In addition, we detected CXCL12 expressing cells in the focal infiltrates, with a significant increase in CXCL12 expression levels in the pSS salivary glands with high FS. Expression of CXCL12 in salivary glands of SS patients have been reported earlier by us and others [12,17,47]. In this study we have extended these results by showing that when dividing pSS patients according to severity of inflammation, different CXCL12 expression pattern appeared.

To our knowledge, we are the first to show that CD138+ plasma cells present in pSS and chronically inflamed salivary glands clearly interact with CXCL12 expressing ductal epithelium and acini and with CXCL12 expressing cells detected in the focal infiltrates. These interactions were by far most abundant in the pSS patients with high FS. CXCL12 has been shown to be vital in the migration and survival of plasma cells [20,48-50] and thus it is possible that also in the inflamed salivary glands these processes are directed by expression of CXCL12.

It is important to keep in mind that in addition to the infiltration of mononuclear cells there is also atrophy, accumulation of adipose tissue and fibrosis in some of the salivary glands from pSS patients. Only a few studies have concerned the role of adipocytes in SS-lesions. Recently, the expression and possible immunoregulatory functions of adipocyte-derived adiponectin have been reported [51,52]. In our study, we describe for the first time that resident adipocytes in the salivary gland tissue of SS patients express CXCL12. This is in accordance with the recognition of adipose tissue as a new player in immune reactions [53]. Indeed, further investigation is needed in order to elucidate interactions between the adipocytes and other elements of the specific milieu of SS [53].

To further examine possible plasma cell survival niches, we studied the expression of IL-6 cytokine. We observed that the expression of IL-6 was highly up-regulated in the salivary glands of pSS patients and increased significantly with increasing FS. Interestingly, there were only a few IL-6 expressing cells in the chronically inflamed tissue and almost no IL-6 expression was detected in glands with normal histology. IL-6 is an inflammatory cytokine that induces anti-apoptotic signals in plasma cells [22]. It is produced by bone marrow stromal cells and is an important factor of bone marrow survival niches. We observed that plasma cells accumulating in the salivary glands of pSS patients interact and are in close proximity to the IL-6 producing cells.

HEVs facilitate migration of lymphocytes into the lymph nodes and Payer's patches by binding certain chemokines such as CCL19, CCL21, CXCL12 and CXCL13 (reviewed in [54]). Interestingly, formation of HEVs has been detected in the inflamed tissue of different autoimmune diseases such as rheumatoid arthritis and SS [35-37]. However, little is known about the properties and contribution of these specialized vascular structures in the migration and retention of cells in the lymphoid compartment of salivary gland tissue of SS patients.

Therefore, by using antibodies against PNAd epitope, we first investigated the expression pattern of HEVs in the salivary glands of pSS patients compared to controls. We detected high numbers of HEVs in pSS patients by comparing with chronically inflamed and normal tissue. In contrast to lymph node HEVs [55], only a few salivary gland HEVs were binding CXCL12. In contrast, CXCL12 chemokines were prominently detected in PNAd negative blood vessels. These results indicate that ectopic HEVs present in the salivary glands of pSS patients have different chemokine expression pattern than HEVs present in secondary lymphoid tissues. Given the demonstrated upregulation of IL-6 expression and selective accumulation of plasma cells in IL-6 rich areas in our analysis, we next investigated whether salivary gland HEVs could bind this factor. A frequent colocalisation between IL-6 and HEVs were detected in the salivary glands of pSS. This finding highlights the potential of HEVs not only to regulate the navigation of lymphoid cells but also their ability to retain important survival factors for plasma cells. Further investigation will shed light into the biological significance of these findings.

## Conclusions

In this initial study, specific factors were identified supporting the presence of plasma cell survival niches in the salivary glands of pSS patients. Survival potential of plasma cells and presence of survival niches increased with severity of the pSS disease (high FS). Our results suggest that plasma cells accumulating in the salivary glands of pSS patients are located in niches rich in CXCL12 and IL-6. Our data do not exclude the existence of other factors important for plasma cell survival in the salivary gland environment. The importance of the complex milieu of pSS salivary glands should be further studied in more detail as our understanding of the local factors necessary for plasma cell survival at the site of inflammation could result in discovery of more selective therapeutic options.

## Abbreviations

AECC: American-European consensus group criteria; ANA: antinuclear antibodies; AP: alkaline phosphatase; CIG: chronically inflamed glands; DAB: diaminobenzidine; FS: focus score; GC: germinal centre; H&E: hematoxylin and eosin; HIER: heat-induced epitope retrieval; HRP: horse radish peroxidase; LPR: liquid permanent red; NG: normal glands; pSS: primary Sjögren's syndrome; RF: rheumatoid factor; RT: room temperature; SS: Sjögren's syndrome; TBS: tris-buffered saline.

## Competing interests

The authors declare that they have no competing interests.

## Authors' contributions

EAS designed and performed the study, analyzed the data and wrote the manuscript. KAB designed the study and proofread the manuscript. GØ performed experiments and wrote the manuscript. MVJ provided the clinical information on the patients and subjects included in the study and proofread the manuscript. RJ initiated the study, organized biopsy collection and supervised and proofread the manuscript. KS designed the study, analyzed the data and wrote the manuscript. All authors read and approved the final manuscript.
